# Prevalence and risk factors of dry eye disease among University Students in Bangkok, Thailand

**DOI:** 10.1371/journal.pone.0258217

**Published:** 2021-10-01

**Authors:** Chantaka Supiyaphun, Passara Jongkhajornpong, Sasivimol Rattanasiri, Kaevalin Lekhanont

**Affiliations:** 1 Department of Ophthalmology, Faculty of Medicine, Vajira Hospital, Navamindradhiraj University, Bangkok, Thailand; 2 Department of Ophthalmology, Faculty of Medicine, Ramathibodi Hospital, Mahidol University, Bangkok, Thailand; 3 Department of Clinical Epidemiology and Biostatistics, Faculty of Medicine, Ramathibodi Hospital, Mahidol University, Bangkok, Thailand; Virginia Commonwealth University, UNITED STATES

## Abstract

**Purpose:**

To investigate the prevalence of dry eye disease (DED) and its associated risk factors among Thai university students.

**Methods:**

A cross-sectional study using an electronic survey was conducted in two Rajabhat universities in Bangkok, Thailand. The woman’s health study questionnaire was used to determine students with DED. The prevalence of DED along with 95% confidence interval (CI) were calculated. Logistic regression model was used to identify the associated risk factors.

**Results:**

A total of 4,111 university students joined and completed the survey questionnaires. Mean age was 18.8 ± 1.1 years with female predominance (2874 students, 69.91%). Clinically diagnosed DED was reported in 136 students (3.31%), while severe symptoms of eye dryness and irritation were reported in 227 students (5.52%). The prevalence of DED among Thai university students was 8.15% (95% CI 7.33% to 9.02%). History of contact lens use and high screen time (> 8 hours per day) were reported in 868 students (21.11%) and 2101 students (51.11%), respectively. Male gender, contact lens use and high screen time were significantly associated with higher risk of DED with the adjusted ORs (95% CI) of 1.39 (1.09, 1.77), 2.49 (1.96, 3.17), and 1.43 (1.14, 1.80), respectively.

**Conclusions:**

DED is not rare among Thai university students. Contact lens use and high screen time are two significant modifiable risk factors of DED in our students. These findings can raise awareness of DED in youth population and provide valuable information for public health promotion in university students.

## Introduction

Dry eye disease (DED) is a multifactorial disease characterized by a loss of homeostasis of tear film and the ocular surface which results in the symptoms of eye irritation, dryness and deterioration of vision [1]. Approximately 5–50% of world’s population is affected by this condition [2]. DED prevalence varies upon different diagnostic criteria, geographical areas, age intervals and gender [2]. Asian countries (e.g. Japan, Korea, Thailand, and China) have been found to have higher prevalence of DED compared to other countries in America and Europe [3–9]. DED is more prevalent in the elderly population, especially amongst women [2]. A recent meta-analysis on DED prevalence in Chinese population demonstrated that age was the only significant covariate with the prevalence of DED by symptoms [10]. However, most of the included studies focused on the adult population aged over 20 years.

To date, the study of DED prevalence in the youth population (age 15–24 years) is still limited. A small amount of studies has been conducted in high school and university students in subtropical and warm temperate countries (i.e. Ghana, Mexico, Japan, and China) [11–15]. The reported DED prevalence was heterogeneous ranging from 10–70.4%. We have previously demonstrated a high prevalence of DED (34%) in hospital-based survey among Thai adults aged 40 years or more [5]. This current study aimed to investigate the prevalence of DED and its associated risk factors in Thai university students using electronic questionnaires.

## Methods

We conducted a cross-sectional study at two Rajabhat universities (Suan Sunandha Rajabhat University; SSRU and Suan Dusit University; SDU) in Bangkok, Thailand, during July 2019 to February 2020. Both universities were composed of six main faculties in Arts and Science including the Faculty of Education, Faculty of Science and Technology, Faculty of Humanities and Social Sciences, Faculty of Industrial Technology, Faculty of Fine Arts, and Faculty of Management Science. This study was conducted under the approval of the Research Ethics Committee of Navamindradhiraj University and Ramathibodi Hospital, Mahidol University (no. MURA 2019/315) in accordance with the tenets of Declaration of Helsinki.

An online survey on dry eye symptoms using the Women’s Health Study Questionnaire (WHS), which was developed by Schaumberg et al. [7]. A self-administered questionnaire was introduced to students by university staffs and via school media advertising. All students who were willing to participate in the survey could access the electronic survey by scanning the QR code linked to the designated mobile Google Form. A total of 4111 students accessed and voluntarily completed the online questionnaire, which took approximately five minutes to complete. The questionnaire consisted of three of the following questions; (1) have you ever been diagnosed by a clinician as having dry eye syndrome? (2) how often do your eyes feel dry (not wet enough)? and (3) how often do your eyes feel irritated? Possible answers to the two questions about symptoms included “constantly,” “often,” “sometimes,” or “never”. DED was determined according to WHS criteria; the presence of either a previous clinical diagnosis of DED or severe symptoms of both dryness and irritation (either constantly or often). Demographics and associated risk factors including history of contact lens use and screen time per day were obtained.

### Statistical analysis

Demographic data and associated risk factors were described using descriptive statistics (i.e. mean along with standard deviation for continuous data and counting numbers with percentage for dichotomous and categorical data) The prevalence of DED and corresponding 95% confidence interval (CI) were calculated.

Logistic regression model was used to identify associated risk factors. Factors with statistical significance from the univariate analysis, were subsequently included in the multivariate analysis. Backward elimination was applied for model selection. Odd ratios (OR) along with 95% CI were estimated to represent a magnitude of the identified associations. Two-tailed P values of less than 0.05 were considered as statistically significantly different. All analyses were calculated using Stata version 16 (StataCorp, College Station, TX, U.S.).

## Results

Of the 4111 students accessed, all students gave online informed consent (100%) and completed the short dry eye questionnaire. 1526 students (37.12%) and 2585 students (62.88%) were from SDU and SSRU, respectively (Table 1). 2874 students (69.91%) were female. The mean age was 18.8 ± 1.1 years. Most of students were studying in the Arts (3373 students, 82.03%) and 359 students (23.53%) were contact lens users. Approximately half of the students (2010 students, 48.89%) had an average screen time over eight hours per day.

**Table 1:** Participant characteristics between 2 universities.

Characteristics	University 1	University 2	Total
	N (%)	N (%)	N (%)
No. of participants	1526 (37.12%)	2585 (62.88%)	4111 (100%)
Gender			
Male	408 (26.74%)	829 (32.07%)	1237 (30.09%)
Female	1118 (73.26%)	1756 (67.93%)	2874 (69.91%)
Mean age (SD)	18.76 (1.15)	18.59 (0.80)	18.65 (0.95)
Faculty			
Science	265 (17.38%)	473 (18.32%)	738 (17.97%)
Arts	1261 (82.62%)	2112 (81.68%)	3373 (82.03%)
Contact lens user	359 (23.53%)	509 (19.69%)	868 (21.11%)
Screen time (hours/day)			
≤ 8	815 (53.41%)	1286 (49.75%)	2101 (51.11%)
> 8	711 (46.59%)	1299 (50.25%)	2010 (48.89%)

### Prevalence of DED among university students

A total of 136 students (3.31%) reported a previous clinical diagnosis of DED, 400 students (9.73%) reported feeling dry eye constantly or often, and 575 students (13.99%) reported feeling irritated eye constantly or often (Table 2). Two hundred and twenty-seven students (5.52%) experiencing both dry eye feeling and eye irritation either constantly or often. Of 4111 students, 335 were classified as having DED according to the definition of WHS criteria. Therefore, the prevalence of DED among Thai university students was estimated at 8.15% (95% CI 7.33% to 9.02%).

**Table 2:** Dry eye questionnaire (N = 4,111 students).

Questionnaire	Yes		No	
1. Have you ever been diagnosed by a clinician as having dry eye syndrome?	136 (3.31%)		3,975 (96.69%)	
**Frequency**	**Constantly**	**Often**	**Sometimes**	**Never**
2. How often do your eyes feel dry (not wet enough)?	33 (0.80%)	367 (8.93%)	2,481 (60.35%)	1,230 (29.92%)
3. How often do your eyes feel irritated?	20 (0.49%)	555 (13.50%)	3,053 (74.26%)	483 (11.75%)

### Risk factors for DED among university students

History of contact lens use and high screen time (> 8 hours per day) were reported in 868 students (21.11%) and 2101 students (51.11%), respectively. Most of students (4028 students, 97.98%) answered the questionnaires during rainy season (July 2019 to mid-October 2019). From univariate analysis, contact lens use and high screen time were significantly associated with higher risk of DED with the ORs (95% CI) of 2.43 (1.92, 3.07) and 1.48 (1.18, 1.86), respectively. After multivariate analysis, we found that male gender, contact lens use and high screen time were significantly associated with higher risk of DED with the adjusted ORs (95% CI) of 1.39 (1.09, 1.77), 2.49 (1.96, 3.17), and 1.43 (1.14, 1.80), respectively (Table 3).

**Table 3:** Risk factors associated with dry eye disease (DED) in university students.

Risk factors	No DED	DED	Odd ratios (95% CI)	P values	Adjusted odd ratios (95% CI)	Adjusted p values
	N = 3776	N = 335				
Contact lens users						
Yes	743 (19.68%)	125 (37.31%)	2.43 (1.92, 3.07)	< 0.001	2.49 (1.96, 3.17)	< 0.001
No	3033 (80.32%)	210 (62.69%)				
Screen time						
> 8 hours/day	1816 (48.09%)	194 (57.91%)	1.48 (1.18, 1.86)	0.001	1.43 (1.14, 1.80)	0.002
≤ 8 hours/day	1960 (51.91%)	141 (42.09%)				
Gender						
Male	1124 (29.77%)	113 (33.73%)	1.20 (0.95,1.52)	0.130	1.39 (1.09, 1.77)	0.008
Female	2652 (70.23%)	222 (66.27%)				
Faculty						
Science	280 (18.11%)	55 (16.42%)	0.89 (0.66, 1.20)	0.441	NA	NA
Arts	3089 (81.89%)	280 (83.58%)				
Seasons						
Winter	75 (1.99%)	8 (2.39%)	1.21 (0.58, 2.52)	0.617	NA	NA
Rainy	3701 (88.01%)	327 (97.61%)				

NA = not applicable

## Discussion

Global prevalence of DED based on the TFOS DEWS II Epidemiology Report ranges from approximately 5% to 50% and varies with the different definition of DED used and the characteristics of the population studied [2]. Based on the results from our large cross-sectional survey, the prevalence of DED among Thai university students was estimated at 8.15% (95% CI 7.33% to 9.02%). The current study used WHS questionnaire which is widely used and accepted for epidemiological study in DED [2]. The major advantage of using this questionnaire is the short survey time and the very high response rate due to a fewer number of questions required. The prevalence of DED in Thai university students (8.15%) appeared to be lower than those of previous studies from Japan and China (21–24%), using the same criteria in similar populations (high school students) [11, 12]. However, the prevalence of clinically diagnosed DED in Thai university students (3.31%) is fairly similar to that of Japanese high school students (4.3% in boys and 8% in girls) [11]. In addition, the prevalence of DED in our study was close to the age-adjusted prevalence reported among US women (7.8%) and the overall prevalence among voluntary participants aged 20–94 years in the Netherlands (9.1%), using the same DED questionnaire (WHS) [7, 16]. The variation of DED prevalence might be occurred due to the differences in study timing and population among the studies. The unmeasurably intrinsic or other extrinsic factors beyond geographical and environmental components such as societal factors, lifestyle activities, and awareness of DED are also important variables affecting the prevalence of DED. Furthermore, the prevalence of DED in young Thai population was less than that reported in Thai adults (34%) [5]. This could be directly explained by the effects of patients’ demographics and diagnostic criteria used.

Two cross-sectional surveys in university students from China and Mexico using the Ocular Surface Disease Index questionnaire (OSDI) found that the prevalence of DED was 10% and as high as 70.4%, respectively [13, 14]. Despite of using the same OSDI questionnaire in similar population, the prevalence between the studies were largely different. We have retrieved all available studies on DED prevalence in youth population from Medline database and have summarized the data in Table 4. Most of studies were conducted in Asia. We observed a high heterogeneity of DED prevalence among different geographical locations, therefore, further well-designed studies with adequate sample size using the same standard questionnaire are warranted for estimating the overall magnitude of DED in the youth population.

**Table 4:** A review of dry eye disease prevalence and risk factors in youth population retrieved from Medline database (last update December 2020).

Authors, Year	Countries/ climate zone	Study settings/ population	Sampling techniques/ dry eye questionnaires	Number of participants	Prevalence of DED	Risk factors for DED
Asiedu K, 2017	Ghana/ tropical zone	Undergraduate students of the University of Cape Coast/ age 18–34 years (mean age 22±2.5 years), female 33.4%, non-CL wearer, non-smoker	Systematic random sampling/SPEED and OSDI^a^	650 out of 700 participants, completed the questionnaire (92.8%)	44.3% (95% CI 40.6%-48.2%)	Over-the-counter eye drop use (OR 4.20, 95% CI 2.61–6.74), any allergies (OR 2.46, 95% CI 1.42–4.29), and oral contraceptives (OR 4.04, 95% CI 1.02–16.01)
						No significant association with sex, alcohol consumption and computer work over one hour.
Garza-León M, 2016	Mexico/ temperate and tropical zones	Students from the University of Monterrey/age 17–33 years (mean age 21.4±1.8 years), female 59.8%, myopia 92%	Stratified sampling according to schools/OSDI	823 out of 860 students (response rate 95.7%)	70.4%	Female (OR 1.29, 95% CI 1.13–1.48), eye drop use (OR 2.00, 95% CI 1.65–2.40), smoking (OR 1.24, 95% CI 1.06–1.46), and hours in front of computer (OR 0.82, 95%CI 0.72–0.93)
						No significant association with CL, and history of refractive surgery.
Li S, 2018	Shanghai, China/ subtropical zone	Freshmen and sophomores on the main campus of Shanghai University/age 18–22 years, female 50%	NA/OSDI & ocular examinations	901 students	10%	A long time eye strain (p = 0.0407) and a use of mobile phones and/or computers for over eight hours daily (p = 0.0129)
						No significant association with sex, age, near work, improper gesture and anxiety.
Uchino M, 2008	Japan/ temperate zone	Private high school students in Tokyo/age 15–18 years, female 25.6%, soft CL users 36.1%, hard CL users 1.7%	NA/WHS	3,433 out of 3,433 students (100% response rate)	NA^b^	CL (OR 4.14, 95% CI 3.42–5.00 in male and OR 4.68, 95% CI 3.02–7.26 in female)
Zhang Y, 2012	Shandong, China/ temperate zone	Senior high school students in Shouguang/ age 15–18 years, female 49.2%	multi-stage stratified random cluster sampling/WHS	1889 out of 1902 students (99.3% response rate), 1885 included in the analysis	23.7%	Inadequate refractive correction (OR 1.98, 95% CI 1.58–2.49), frequent self-administered topical ophthalmic medications (OR 1.84, 95% CI 1.40–2.41), and poor sleep quality (OR 1.34, 95% CI 1.05–1.71)
						No significant association with sex, myopia, and CL use.

a. OSDI ≥13 and SPEED ≥6 were used to defined symptomatic dry eye.

b. Clinically diagnosed dry eye disease was present in 123 boys (4.3%) and 47 girls (8.0%). Severe symptoms of dry eye disease were observed in 599 subjects in boys (21.0%) and 143 in girls (24.4%).

**Abbreviations:** CI = confidence interval, CL = contact lens, DED = dry eye disease, NA = not available, OR = odds ratio, OSDI = Ocular Surface Disease Index, SPEED = Standardized Patient Evaluation of Eye Dryness, WHS = Women’s Health Study.

Several risk factors for DED are consistently identified, both non-modifiable (e.g. aging, female gender, and Asian race) and modifiable factors (e.g. computer use, contact lens wear, environment, and medication) [2]. This current study found a slightly higher proportion of DED in male (9.13%) compared to female (7.72%) (adjusted OD 1.39 [95% CI 1.09, 1.77], adjusted p-value = 0.008), which was in the same trend observed among high school students in Shangdong, China (51.7% in male and 48.3% in female, p-value = 0.234) [12]. This might be explained by environmental and lifestyle factors. Men tend to participate in outdoor activities more frequently than women, exposing them to a variety of environmental stresses such as air pollution, smoke, desiccating wind, intense ultraviolet and extreme temperatures. In addition, our finding supports the previous systematic review which demonstrated inconsistent sex differences in the prevalence of DED at ages lower than 40 years, though the female gender becomes a consistent factor associated with DED at ages above 50 years [2]. Theoretically, sex-related difference in DED prevalence is mainly attributed to the effects of sex steroids (e.g. androgens and estrogens), hypothalamic-pituitary hormones, glucocorticoids, insulin, insulin-like growth factor 1 and thyroid hormones, as well as to the sex-specific genetic and epi-genetic factors [17]. Moreover, more prevalence of dry eye associated systemic conditions was also found in female compared to male, such as autoimmune disorders, functional disorders, atopic diseases and allergy, and most psychiatric disorders [16, 17]. The present study was conducted in young and healthy population (university students), therefore this might be another reason to support why the association of female on DED in young population was not prominent.

Climatic and environmental changes have differential adverse impacts on dry eye and likely occur in tropical countries where sunlight and wind exposure is immense [2, 18–20]. Previous evidence showed that low humidity (< 40%) increased tear evaporation and subsequently worsened dry eye symptoms [21]. On the other hand, increasing local humidity could improve dry eye symptoms through the mechanism of increasing tear lipid layer thickness [22, 23]. Additionally, tear evaporation rate was reduced to near zero at 70% humidity [21]. Thailand has a tropical climate with 3 distinct seasons; 1) rainy or southwest monsoon season (mid-May to mid-October), 2) winter or northeast monsoon season (mid-October to mid-February), and 3) summer or pre-monsoon season (mid-February to mid-May). Nevertheless, Bangkok is located in the central part of Thailand, where temperature (25.9°C—28.0°C) and humidity (63% - 81%) are constantly high throughout the study period even the season changes [24]. Therefore, we did not observe a significant association between seasons and DED in our population.

Contact lenses divide the tear film into two layers (i.e. pre- and post-lens tear film). This change can lead to instability and thinning of pre- and post-lens tear film, resulting in ocular dryness and increased friction between the contact lens and the ocular surface [25]. We also found that contact lens use associated with the higher risk of DED at the OR of 2.5, corresponding to the results from Uchino M et al. studying in Japanese high school students [11]. Screen time per day was another modifiable factor of DED, which was identified from our study. Students who used digital screens for more than eight hours per day were significantly associated with DED at the OR of 1.4 compared to those who used digital screen equal or less than eight hours per day, which was similar to the report in university students in Shanghai, China by Li S et al. [26]. It has been proven that using digital screen create blink abnormalities (incomplete blink and reduced blink rate) [27, 28]. These prolonged situations possibly contribute to tear film instability, epithelial damage, and symptomatic dry eye. Additionally, damaging high-energy visible blue light and the presence of inflammation found on the ocular surface of visual display terminal (VDT) users are considered as other mechanisms of VDT-associated DED [29].

This study has several limitations. Participants were voluntarily recruited from two Rajabhat universities in Bangkok. Their socioeconomic status, lifestyle and environment might be different from those in other universities in different locations. Additionally, as QR code scanning was required for accessing to the questionnaire, students without smartphones could not participate in this survey. These selection biases could interfere our estimated DED prevalence. Some possible factors of DED including smoking status, underlying diseases and medications were not considered. However, we expected that the prevalence of these omitted factors to be very low in our target population because smoking is prohibited in every university and the students in this age range are generally healthy.

## Conclusion

In summary, DED among Thai university students is not uncommon. Based on the current large survey, approximately 8 in 100 students are affected by this condition. Contact lens use and high screen time of over eight hours are two significant modifiable risk factors of DED in Thai university students. Our findings can raise awareness of DED in youth population and provide valuable information for public health promotion among university students.

## Supporting information

S1 File (XLS)

## References

[1] Craig JP, Nichols KK, Akpek EK, Caffery B, Dua HS, Joo CK, et al. TFOS DEWS II Definition and Classification Report. Ocul Surf. 2017;15(3):276–83. doi: 10.1016/j.jtos.2017.05.008 28736335

[2] Stapleton F, Alves M, Bunya VY, Jalbert I, Lekhanont K, Malet F, et al. TFOS DEWS II Epidemiology Report. Ocul Surf. 2017;15(3):334–65. doi: 10.1016/j.jtos.2017.05.003 28736337

[3] Uchino M, Nishiwaki Y, Michikawa T, Shirakawa K, Kuwahara E, Yamada M, et al. Prevalence and risk factors of dry eye disease in Japan: Koumi study. Ophthalmology. 2011;118(12):2361–7. doi: 10.1016/j.ophtha.2011.05.029 21889799

[4] Ahn JM, Lee SH, Rim TH, Park RJ, Yang HS, Kim TI, et al. Prevalence of and risk factors associated with dry eye: the Korea National Health and Nutrition Examination Survey 2010–2011. Am J Ophthalmol. 2014;158(6):1205–14.e7. doi: 10.1016/j.ajo.2014.08.021 25149910

[5] Lekhanont K, Rojanaporn D, Chuck RS, Vongthongsri A. Prevalence of dry eye in Bangkok, Thailand. Cornea. 2006;25(10):1162–7. doi: 10.1097/01.ico.0000244875.92879.1a 17172891

[6] Jie Y, Xu L, Wu YY, Jonas JB. Prevalence of dry eye among adult Chinese in the Beijing Eye Study. Eye (Lond). 2009;23(3):688–93. doi: 10.1038/sj.eye.6703101 18309341

[7] Schaumberg DA, Sullivan DA, Buring JE, Dana MR. Prevalence of dry eye syndrome among US women. Am J Ophthalmol. 2003;136(2):318–26. doi: 10.1016/s0002-9394(03)00218-6 12888056

[8] Schaumberg DA, Dana R, Buring JE, Sullivan DA. Prevalence of dry eye disease among US men: estimates from the Physicians’ Health Studies. Arch Ophthalmol. 2009;127(6):763–8. doi: 10.1001/archophthalmol.2009.103 19506195 PMC2836718

[9] Viso E, Rodriguez-Ares MT, Gude F. Prevalence of and associated factors for dry eye in a Spanish adult population (the Salnes Eye Study). Ophthalmic Epidemiol. 2009;16(1):15–21. doi: 10.1080/09286580802228509 19191177

[10] Song P, Xia W, Wang M, Chang X, Wang J, Jin S, et al. Variations of dry eye disease prevalence by age, sex and geographic characteristics in China: a systematic review and meta-analysis. J Glob Health. 2018;8(2):020503. doi: 10.7189/jogh.08.020503 30206477 PMC6122008

[11] Uchino M, Dogru M, Uchino Y, Fukagawa K, Shimmura S, Takebayashi T, et al. Japan Ministry of Health study on prevalence of dry eye disease among Japanese high school students. Am J Ophthalmol. 2008;146(6):925–9.e2. doi: 10.1016/j.ajo.2008.06.030 18723141

[12] Zhang Y, Chen H, Wu X. Prevalence and risk factors associated with dry eye syndrome among senior high school students in a county of Shandong Province, China. Ophthalmic Epidemiol. 2012;19(4):226–30. doi: 10.3109/09286586.2012.670742 22650150

[13] Li S, He J, Chen Q, Zhu J, Zou H, Xu X. Ocular surface health in Shanghai University students: a cross-sectional study. BMC Ophthalmol. 2018;18(1):245. doi: 10.1186/s12886-018-0825-z 30208892 PMC6134707

[14] Garza-León M, Valencia-Garza M, Martínez-Leal B, Villarreal-Peña P, Marcos-Abdala HG, Cortéz-Guajardo AL, et al. Prevalence of ocular surface disease symptoms and risk factors in group of university students in Monterrey, Mexico. J Ophthalmic Inflamm Infect. 2016;6(1):44. doi: 10.1186/s12348-016-0114-z 27864795 PMC5116015

[15] Asiedu K, Kyei S, Boampong F, Ocansey S. Symptomatic Dry Eye and Its Associated Factors: A Study of University Undergraduate Students in Ghana. Eye Contact Lens. 2017;43(4):262–6. doi: 10.1097/ICL.0000000000000256 26963438

[16] Vehof J, Snieder H, Jansonius N, Hammond CJ. Prevalence and risk factors of dry eye in 79,866 participants of the population-based Lifelines cohort study in the Netherlands. Ocul Surf. 2020. Epub 2020/05/08. doi: 10.1016/j.jtos.2020.04.005 32376389

[17] Sullivan DA, Rocha EM, Aragona P, Clayton JA, Ding J, Golebiowski B, et al. TFOS DEWS II Sex, Gender, and Hormones Report. Ocul Surf. 2017;15(3):284–333. doi: 10.1016/j.jtos.2017.04.001 28736336

[18] Alves M, Novaes P, Morraye Mde A, Reinach PS, Rocha EM. Is dry eye an environmental disease? Arq Bras Oftalmol. 2014;77(3):193–200. doi: 10.5935/0004-2749.20140050 25295911

[19] Berg EJ, Ying GS, Maguire MG, Sheffield PE, Szczotka-Flynn LB, Asbell PA, et al. Climatic and Environmental Correlates of Dry Eye Disease Severity: A Report From the Dry Eye Assessment and Management (DREAM) Study. Transl Vis Sci Technol. 2020;9(5):25. doi: 10.1167/tvst.9.5.25 32821497 PMC7401914

[20] Mandell JT, Idarraga M, Kumar N, Galor A. Impact of Air Pollution and Weather on Dry Eye. J Clin Med. 2020;9(11). doi: 10.3390/jcm9113740 33233863 PMC7699870

[21] Madden LC, Tomlinson A, Simmons PA. Effect of humidity variations in a controlled environment chamber on tear evaporation after dry eye therapy. Eye Contact Lens. 2013;39(2):169–74. doi: 10.1097/ICL.0b013e318283dfc6 23411993

[22] Korb DR, Greiner JV, Glonek T, Esbah R, Finnemore VM, Whalen AC. Effect of periocular humidity on the tear film lipid layer. Cornea. 1996;15(2):129–34. doi: 10.1097/00003226-199603000-00004 8925659

[23] Starr CE, Dana R, Pflugfelder SC, Holland EJ, Zhang S, Owen D, et al. Dry eye disease flares: A rapid evidence assessment. Ocul Surf. 2021;22:51–9. doi: 10.1016/j.jtos.2021.07.001 34303844

[24] Climate Bangkok (Thailand). Weather by Month // Weather Averages Bangkok. Available from: https://en.climate-data.org/asia/thailand/bangkok/bangkok-6313/ accessed 8 August 2021

[25] Kojima T. Contact Lens-Associated Dry Eye Disease: Recent Advances Worldwide and in Japan. Invest Ophthalmol Vis Sci. 2018;59(14):Des102–des8. doi: 10.1167/iovs.17-23685 30481813

[26] Li J, Zheng K, Deng Z, Zheng J, Ma H, Sun L, et al. Prevalence and risk factors of dry eye disease among a hospital-based population in southeast China. Eye Contact Lens. 2015;41(1):44–50. doi: 10.1097/ICL.0000000000000064 25232992

[27] Doughty MJ. Consideration of three types of spontaneous eyeblink activity in normal humans: during reading and video display terminal use, in primary gaze, and while in conversation. Optom Vis Sci. 2001;78(10):712–25. doi: 10.1097/00006324-200110000-00011 11700965

[28] Freudenthaler N, Neuf H, Kadner G, Schlote T. Characteristics of spontaneous eyeblink activity during video display terminal use in healthy volunteers. Graefes Arch Clin Exp Ophthalmol. 2003;241(11):914–20. doi: 10.1007/s00417-003-0786-6 14586592

[29] Mehra D, Galor A. Digital Screen Use and Dry Eye: A Review. Asia Pac J Ophthalmol (Phila). 2020. doi: 10.1097/APO.0000000000000328 33181547

